# Purine and purinergic receptors in health and disease

**DOI:** 10.1002/mco2.359

**Published:** 2023-09-07

**Authors:** Yanling Ai, Hengyi Wang, Lu Liu, Yulin Qi, Shiyun Tang, Jianyuan Tang, Nianzhi Chen

**Affiliations:** ^1^ Department of Oncology Hospital of Chengdu University of Traditional Chinese Medicine Chengdu China; ^2^ Department of Infectious Diseases Hospital of Chengdu University of Traditional Chinese Medicine Chengdu China; ^3^ School of Pharmacy Chengdu University of Traditional Chinese Medicine Chengdu China; ^4^ Department of Ophthalmology The First Affiliated Hospital of Guangzhou University of Chinese Medicine Guangzhou University of Chinese Medicine Guangzhou China; ^5^ Postdoctoral Research Station of Guangzhou University of Chinese Medicine Guangzhou China; ^6^ Hospital of Chengdu University of Traditional Chinese Medicine Chengdu China; ^7^ TCM Regulating Metabolic Diseases Key Laboratory of Sichuan Province Hospital of Chengdu University of Traditional Chinese Medicine Chengdu China; ^8^ State Key Laboratory of Ultrasound in Medicine and Engineering College of Biomedical Engineering Chongqing Medical University Chongqing China

**Keywords:** ATP, cancer, immunity, P2x7, purinergic, tumor microenvironment

## Abstract

Purines and purinergic receptors are widely distributed throughout the human body. Purine molecules within cells play crucial roles in regulating energy metabolism and other cellular processes, while extracellular purines transmit signals through specific purinergic receptors. The ubiquitous purinergic signaling maintains normal neural excitability, digestion and absorption, respiratory movement, and other complex physiological activities, and participates in cell proliferation, differentiation, migration, and death. Pathological dysregulation of purinergic signaling can result in the development of various diseases, including neurodegeneration, inflammatory reactions, and malignant tumors. The dysregulation or dysfunction of purines and purinergic receptors has been demonstrated to be closely associated with tumor progression. Compared with other subtypes of purinergic receptors, the P2X7 receptor (P2X7R) exhibits distinct characteristics (i.e., a low affinity for ATP, dual functionality upon activation, the mediation of ion channels, and nonselective pores formation) and is considered a promising target for antitumor therapy, particularly in patients with poor response to immunotherapy This review summarizes the physiological and pathological significance of purinergic signaling and purinergic receptors, analyzes their complex relationship with tumors, and proposes potential antitumor immunotherapy strategies from tumor P2X7R inhibition, tumor P2X7R overactivation, and host P2X7R activation. This review provides a reference for clinical immunotherapy and mechanism investigation.

## Graphical abstract

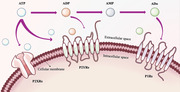

## 1 INTRODUCTION

The significant advancements in social and economic development have led to notable transformations in the health conditions, lifestyle patterns, and dietary structures of residents. Consequently, there has been a remarkable increase in global life expectancy from 66.8 years in 2000 to 73.3 years in 2019.^1^ As the mortality of infectious diseases has declined, noncommunicable diseases, including neoplasms, have emerged as a new generation of health risk factors. With the constantly evolving spectrum of diseases and increasingly severe public health challenges, researchers not only focus on the symptomatic treatment of pathological products and abnormal manifestations but also strive to comprehend the behavior patterns of various biological molecules in physiological or pathological states from the perspective of human function and metabolism. By elucidating the fundamental principles and shared mechanisms of biological molecules in both healthy and diseased states, potentially feasible solutions can be proposed for disease prevention and treatment.

Purine and its derivatives, exemplified by adenosine 5′‐triphosphate (ATP) and adenosine, have garnered increasing attention among various biological molecules. The indispensable role of ATP as a fuel source for the robust growth of organisms has long been recognized. In the 1930s, Drury and Szent‐Gyorgyi published a seminal report on the impact of adenine nucleotides on mammalian hearts, providing an initial insight into the physiological activity of purines and purinergic signaling.^2^ Subsequently, it was found that in addition to regulating energy metabolism and other processes within cells, purine molecules can also bind to extracellular purinergic receptors, stimulate signaling pathways, and exert specific functions.^3^ In the 1970s, the “purinergic” hypothesis was proposed and developed,^4^, ^5^, ^6^ and then research on the role of purinergic signaling under different conditions has been extensively conducted.^7^ Purinergic signaling is associated with other signaling molecules to participate in various physiological and pathological processes such as development, aging, regeneration, and inflammation. At present, the conversion of research findings into tailored drugs based on clinical demands has emerged as a prevailing trend, and the intervention in tumors based on purinergic signaling has been gradually highlighted.

Numerous studies have demonstrated a strong association between dysfunction of the purine/purinergic system and the onset of various diseases.^8^, ^9^ In terms of cause‐specific disability‐adjusted life years, the burden of cancer surpasses that of all other human diseases in clinical, social, and economic aspects.^10^ The existing studies have shown that the incidence and mortality of cancer are on the rise due to the aging population and the prevalence of carcinogenic factors.^11^ According to the statistics of the World Health Organization,^12^ the top four leading causes of death before the age of 70 years are tumors in nearly 74% of 183 countries. The formation of tumors is a complex multistep process, which is the result of the interaction between internal genetic factors and external physical, chemical, and biological factors. With the deepening of research, the metabolism and regulation of various components in the tumor microenvironment (TME) also provide new therapeutic ideas for delaying tumor deterioration and improving immunotherapy.^13^, ^14^ Multiple biomolecules, including purines and purinergic receptors, play a crucial role in regulating the malignant phenotype during tumor progression. Among these biomolecules, the P2X7 receptor (P2X7R) stands out due to its unique structure and function. Compared with other subtypes, P2X7R has a low sensitivity to ATP and possesses the ability not only to mediate ion channels but also to form nonselective large pores upon activation, thereby exerting bidirectional regulatory effects.^15^ Additionally, a large number of studies have shown that P2X7R in living organisms exhibits obvious oncogene‐like characteristics, and its abnormal expression and function are closely related to the occurrence and development of a variety of tumors.^16^, ^17^ Blockade or overstimulation of P2X7R has been proven to have an inhibitory effect on tumor cells and is a potential antitumor target.^18^, ^19^

The review provides a comprehensive overview of the fundamental functions of purines and purinergic receptors in both physiological and pathological conditions. Specifically, the impact of purines and purinergic receptors on tumor progression is analyzed, with particular emphasis on the expression, function, and characteristics of P2X7R in tumors. In view of the immunological characteristics of the TME and the current challenges of immunotherapy, the treatment direction and clinical application prospects of targeting P2X7R are discussed to provide a reference for clinical treatment and mechanism research.

## 2 OVERVIEW OF PURINES AND PURINERGIC RECEPTORS

### 2.1 The duality of ATP

It is widely acknowledged that the progression of life activities inherently involves the acquisition, storage, release, utilization, and dissipation of energy. The ATP, consisting of adenosine and three phosphate groups, facilitates efficient and dynamic utilization and transformation of energy, thereby promoting the smooth progression of metabolism. In addition to being a universal energy currency in the biological realm, ATP is also an important extracellular signaling molecule.^20^ In fact, the purinergic signaling system has been present since the early stages of biological evolution.^21^ ATP is expressed ubiquitously across various types of tissues and cells. In the organism, it mediates neurotransmission, transduces mechanosensation, regulates vascular function, and also participates in biological behavioral regulation, such as cell proliferation, differentiation, migration, and death.^22^ For example, the ATP stored in synaptic vesicles can act as a neurotransmitter in neurons, facilitating the transmission of excitation through exocytosis.^23^ Once entering the extracellular space, nucleotides such as ATP will be degraded by different extracellular nucleotidases into corresponding nucleosides.^24^ For instance, nucleoside triphosphate dephosphorylase (CD39) can convert ATP or adenosine diphosphate (ADP) into adenosine monophosphate (AMP), while ecto‐5′‐nucleotidase (CD73) further converts AMP into adenosine.^25^ The abnormal metabolism of extracellular ATP (eATP) not only represents a timely response to various cellular stressors such as inflammation and tissue damage,^26^ but also exerts critical effects on the development and immune regulation of the TME.^27^, ^28^

### 2.2 Classification of purinergic receptors

The eATP facilitates signal transduction through specific plasma membrane receptors.^8^ In 1978, Geoffrey Burnstock used the term “purinoceptors” to define a class of protein receptors expressed on the cell surface. Based on their structural and functional characteristics, purinergic receptors are classified into two major categories: P1 receptors (P1Rs) and P2 receptors (P2Rs). P1Rs refer to a class of G protein‐coupled receptors (GPCRs) that are primarily activated by the ATP metabolite adenosine. Depending on their subtype, the P1Rs can be further categorized into A1 receptor (A1R), A2A receptor (A2AR), A2B receptor (A2BR), and A3 receptor (A3R).^29^ The activation of P2Rs can be induced by ATP or other nucleotides, and P2Rs are divided into P2X receptors (P2XRs) and P2Y receptors (P2YRs).^30^ P2XRs are functionally assembled as trimeric channels and there are seven known subtypes of P2X1‐7R. eATP is the physiological activator of P2XRs. The opening of P2XRs allows a substantial influx of Ca^2+^ and Na^+^ and efflux of K^+^, resulting in an elevation in intracellular Ca^2+^ concentration, excessive accumulation of Na^+^, and depletion of K^+^ .^31^ Additionally, P2YRs consist of eight recognized subtypes: P2Y1R, P2Y2R, P2Y4R, P2Y6R, P2Y11R, P2Y12R, P2Y13R, and P2Y14R.^32^ The absent numerical values indicate nonmammalian orthologs or receptors lacking any empirical support for nucleotide responsiveness.

### 2.3 Structure of purinergic receptors

Different types of purinergic receptors possess distinct structural characteristics. P1Rs are coupled to adenylyl cyclase, where A1R and A3R are negatively coupled, while A2AR and A2BR are positively coupled to adenylyl cyclase.^30^ The A1R and A3R receptors exhibit a preference for coupling to Gαi/o based on sequence similarity and G protein coupling specificity, whereas the A2AR and A2BR receptors display a greater inclination toward Gαs.^33^ P2XRs are ligand‐gated ion channels that are composed of a substantial extracellular loop containing 10 conserved cysteine residues, two transmembrane domains (TM1 is involved in channel gating, while TM2 forms the ion pore), and intracellular N‐ and C‐terminal subunit topologies.^32^ The mammalian P2XRs can form either homomeric or heteromeric trimeric channels (the shortest length of the P2X4R protein is 388, while the longest length of the P2X7 protein is 595), and each channel is composed of three ATP binding sites.^34^ Structurally, P2X7R has an extremely long intracellular C‐terminus, constituting 40% of the entire protein. This feature is widely regarded as the primary determinant of its physiological function.^35^ The P2YRs belong to the δ subgroup of class A, rhodopsin‐like GPCRs. Upon activation, P2YRs couple with heterotrimeric G proteins. Among these receptors, the P2Y1, P2Y2, P2Y4, P2Y6, and P2Y11 subtypes couple with Gαq/11, while the P2Y12, P2Y13, and P2Y14 receptors couple with Gαi/o. Additionally, the P2Y11 receptor couples with Gαs.^36^ Purinergic receptors are widely distributed throughout the human body, and their structural diversity is the foundation for regulating a multitude of physiological and pathological processes, thus holding significant importance in various life activities.

## 3 RELATIONSHIP BETWEEN PURINERGIC SIGNALING AND ORGANISM

### 3.1 Transmission and regulatory mechanisms of purinergic signaling

**FIGURE 1:**
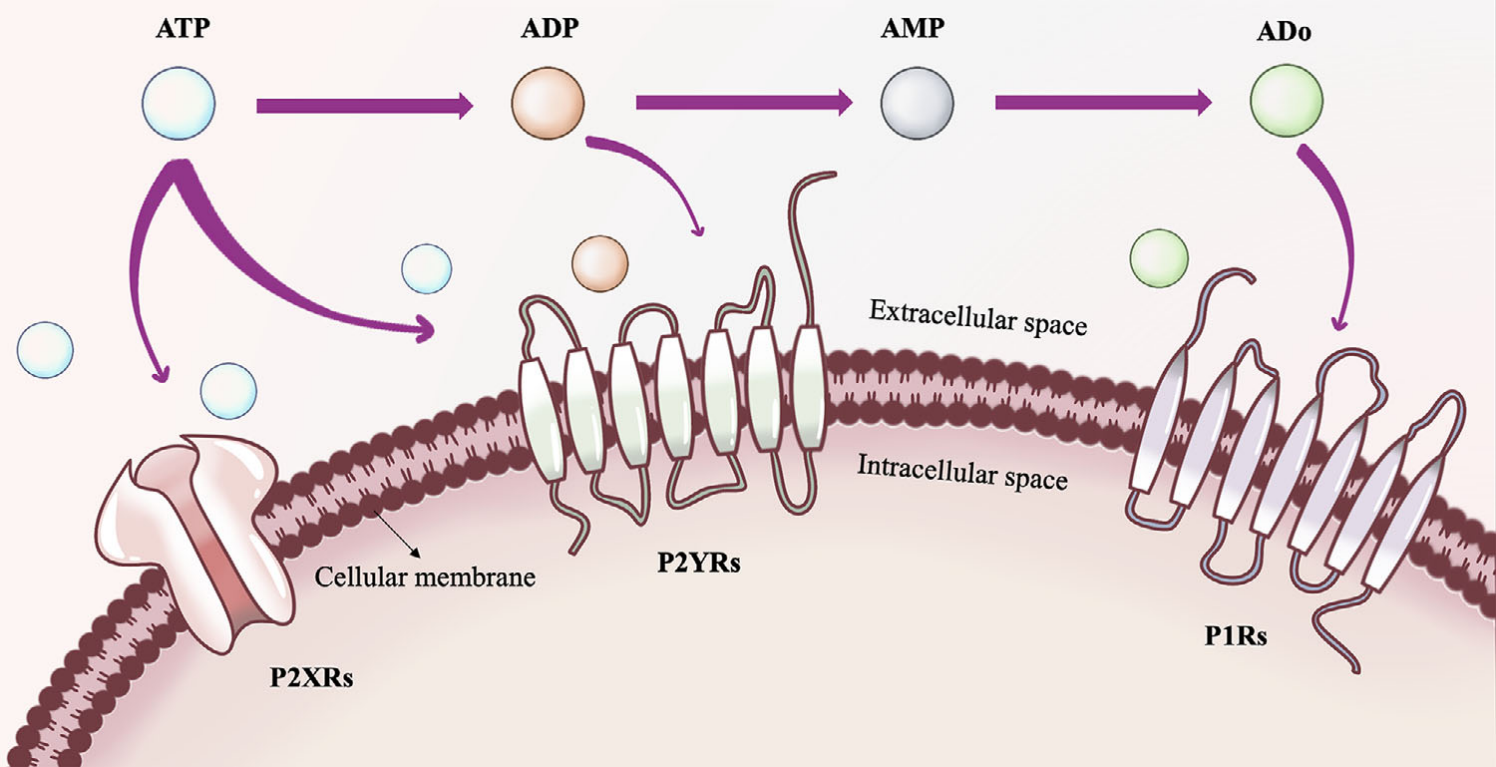
Example diagram of purine signaling pathway. ATP, adenosine 5′‐triphosphate; AMP, adenosine monophosphate; Ado, adenosine; P1Rs, P1 receptors; P2XRs, P2X receptors; P2YRs, P2Y receptors.

Purinergic signaling serves as a ubiquitous intercellular communication mediated by extracellular nucleotides and nucleosides, which is a multistep and evolutionarily conserved cascade reaction (Figure 1).^37^, ^38^ First, the endogenous nucleotides are released and subsequently metabolized to the extracellular environment.^39^ Second, extracellular nucleotides selectively bind to either P1Rs or P2Rs, thereby exerting distinct biological effects. After signal transduction, extracellular nucleotides are degraded or inactivated through membrane binding and the action of soluble nucleotidases such as CD73, ecto‐nucleoside triphosphate diphosphohydrolase, ecto‐nucleotide pyrophosphatase/phosphodiesterase, and alkaline phosphatases.^40^ Finally, the resultant adenosine binds to its own receptors and undergoes metabolism via adenosine deaminase and purine nucleoside phosphorylases, or is internalized by cells.^41^ The initial research on purinergic signaling mainly focused on short‐term transmission such as neurotransmission, neural regulation, and chemotaxis. In recent years, the long‐term signal transduction pathways involving cellular proliferation, differentiation, migration, and apoptosis have also received increasing attention.^30^

### 3.2 Association between purinergic signaling and health

The physiological effects of purines and purinergic receptors are diverse and complex. Purine compounds, including ATP, ADP, and adenosine, actively participate in cellular energy metabolism while also serving as intercellular signaling molecules. Purinergic receptors are widely distributed and expressed in tissues, playing a crucial role in the regulation of diverse physiological functions through their interaction with purine compounds. For instance, the expression of purinergic receptors on neurons and glial cells in the nervous system can exert regulatory control over neurotransmitter release and neuronal excitability.^42^ In the cardiovascular system, purinergic receptors modulate the contraction and relaxation of the heart and blood vessels by acting on cardiac muscle cells, vascular smooth muscle cells, and endothelial cells.^42^ Within the digestive system, purinergic receptors expressed on intestinal smooth muscle cells and enteric neurons regulate gastrointestinal motility, the secretion of digestive fluids, and intestinal absorption.^43^ Within the respiratory system, purinergic receptors maintain airway and alveolar homeostasis in bronchial epithelial cells, alveolar epithelial cells, and smooth muscle cells.^44^, ^45^ In fact, purinergic signal transduction is involved in a series of complex events throughout the entire lifespan.

### 3.3 Association between purinergic signaling and diseases

Given the critical role of purinergic signaling in fundamental metabolic processes, initial research focused mainly on its physiological and biochemical functions. However, as practical knowledge has accumulated, greater emphasis has been placed on exploring the pathophysiology and therapeutic potential of purines and purinergic receptors across diverse diseases.

#### 3.3.1 Association between P1Rs and diseases

P1Rs are primarily located in the heart, lungs, kidneys, and central nervous system, where they regulate adenylyl cyclase activity and exert anti‐inflammatory, analgesic, and sedative effects^33^ (Table 1). The four subtypes of receptors exhibit varying affinities for adenosine. In general, A1R, A2AR, and A3R receptors present a relatively high affinity for adenosine and can be activated at the nanomolar (nM) level. The activation of A2B requires a higher concentration of adenosine at the micromolar (μM) level, which may potentially be linked to tissue damage caused by inflammation, ischemia, or hypoxia.^46^ Adenosine is an endogenous purine nucleoside with cardioprotective properties, and the role of P1Rs in cardiovascular diseases has garnered significant attention. Studies have shown that impaired P1 receptor function results in blocked lipid transport and platelet aggregation, leading to the development of atherosclerosis and myocardial infarction.^47^ The occurrence of arrhythmia, myocardial ischemia–reperfusion injury, angina pectoris, chronic heart failure, and other diseases is also closely associated with P1Rs.^48^ Furthermore, the P1Rs play a crucial role in the pathogenesis of neurological disorders. These receptors, particularly A1R and A2AR, are prominently expressed in the brain and serve as vital mediators for adenosine regulation and integration of cerebral function.^49^ The utilization of specific drugs to activate or inhibit P1Rs represents a therapeutic approach for central nervous system disorders, including epilepsy, pain, affective disorders, Parkinson's disease, Alzheimer's disease, and cerebral ischemia.^50^

**TABLE 1:** A partial list of clinically approved adenosine receptor drugs.

Mechanism of action	Name	Therapeutic use
A1R agonist	Adenosine	Tachycardia
	Caffeine	Respiration disorders
	Adenine	Alcoholism, leukemia
A1R antagonist	Theophylline	Lung diseases, emphysema, asthma, airway obstruction, asthmatic bronchitis, chronic bronchitis
	Proxyphylline	Asthma, heart failure, asthmatic bronchitis
	Doxofylline	Asthma, bronchitis
	Bamifylline	Asthma
	Acotiamide hydrochloride hydrate	Dyspepsia
	Caffeine citrate	Apnea
	Theobromin	Cardiovascular diseases
	Theophylline sodium glycinate	Asthma, pulmonary edema, bronchitis
A2AR agonist	Adenosine	Myocardial perfusion imaging
	Regadenoson	Contrast agents, myocardial ischemia
	Adenine	Alcoholism, leukemia
A2AR antagonist	Istradefylline	Parkinson's disease
	Caffeine	Respiration disorders
	Theobromin	Cardiovascular diseases
	Proxyphylline	Asthma, heart failure, asthmatic bronchitis
A2BR antagonist	Theophylline	Lung diseases, emphysema, asthma, airway obstruction, asthmatic bronchitis, chronic bronchitis
	Theophylline sodium glycinate	Asthma, pulmonary edema, bronchitis
A3R agonist	Adenine	Alcoholism, leukemia

#### 3.3.2 Association between P2XRs and diseases

The presence of P2XRs is predominantly observed in the nervous and immune systems, and structural abnormalities in these receptors may underlie pathological functions such as pain, inflammatory bone loss, immune response deficiency, and male infertility.^51^ P2X1R is mainly expressed on platelets and neutrophils, contributing to platelet activation and thrombus formation.^52^ Deficiency in P2X1R may lead to inflammation‐induced damage to intestinal vascular integrity and severe hemorrhaging.^53^ Additionally, P2X1R is also implicated in bladder dysfunction.^54^ P2X2R and P2X3R play a role in regulating somatic sensation, and their antagonists have the potential to abolish responses to taste stimuli.^55^ P2X2R can protect hearing, and its dysfunction can lead to hearing impairment.^56^ P2X3R is a promising therapeutic target for refractory chronic cough of unknown etiology.^57^ P2X4R represents a potential target for central nervous system disorders such as chronic pain, epilepsy, local ischemia, multiple sclerosis, and neurodegenerative diseases.^58^, ^59^ P2X5R is highly expressed in the mature stage of osteoclasts, and is an important regulator of inflammatory‐related bone loss and osteoclast multinucleation, as well as a therapeutic target for inflammation‐related bone loss.^60^, ^61^ P2X6R is involved in inflammation and immune regulation.^62^ Compared with other P2XRs, P2X7R has a lower affinity for ATP under normal physiological concentrations,^63^ while it can still be activated in pathological conditions, thereby contributing to the development of neurological diseases, cardiovascular diseases, malignant tumors, and many proinflammatory events.^64^, ^65^, ^66^

#### 3.3.3 Association between P2YRs and diseases

P2YRs mainly exist in the cardiovascular, immune, musculoskeletal, and nervous systems and are associated with pathological processes such as thrombosis, the release of inflammatory factors, and osteoporosis.^67^ First, P2YRs are ubiquitously expressed in all blood cells, such as erythrocytes, platelets, monocytes, and granulocytes, playing pivotal roles as regulatory factors in the pathogenesis of cardiovascular diseases.^68^ Studies have shown that P2Y1R, P2Y2R, and P2Y6R exert a crucial effect on the pathogenesis of acute vascular inflammation and atherosclerosis,^69^, ^70^, ^71^ while P2Y1R and P2Y12R selectively facilitate platelet aggregation and thrombus formation,^72^, ^73^ thereby presenting themselves as potential targets for drug therapy. Second, P2YRs also play an important role in the immune system. Specifically, P2Y2R, P2Y6R, P2Y12R, and P2Y14R have been implicated in the pathogenesis of various inflammatory diseases characterized by acute onset, chronic progression, and fibrosis.^74^, ^75^ P2Y12R provides a novel therapeutic target for autoimmune diseases.^76^ Osteoporosis is a chronic disease that poses a significant threat to bone health. P2Y2R, P2Y6R, P2Y12R, and P2Y13R are involved in this detrimental process, and the development of corresponding antagonists may represent an advantageous preventive and therapeutic strategy.^77^, ^78^, ^79^ In addition, P2YRs also play a crucial role in the nervous system. The activation of P2Y1R and P2Y12R can exert neuroprotective effects and have important implications for recovery from cerebral infarction and neurodegenerative diseases.^80^

Abnormal expression or dysfunction of purines and purinergic receptors can give rise to the occurrence and progression of diverse pathological conditions. Therefore, conducting in‐depth research on purines and purinergic receptors can facilitate the identification of novel therapeutic targets and drugs, providing more effective approaches for clinical treatment.^81^ The impact of purinergic signaling on specific diseases has been extensively discussed in numerous articles.^8^, ^9^ Therefore, this review primarily focuses on the correlation between purines/purinergic receptors and tumors, with particular emphasis on the pivotal role played by the receptor P2X7R in tumor progression, immunotherapy characteristics, and prospects for clinical application.

## 4 PURINES AND PURINERGIC RECEPTORS IN TUMORS

### 4.1 Tumor and TME

**FIGURE 2:**
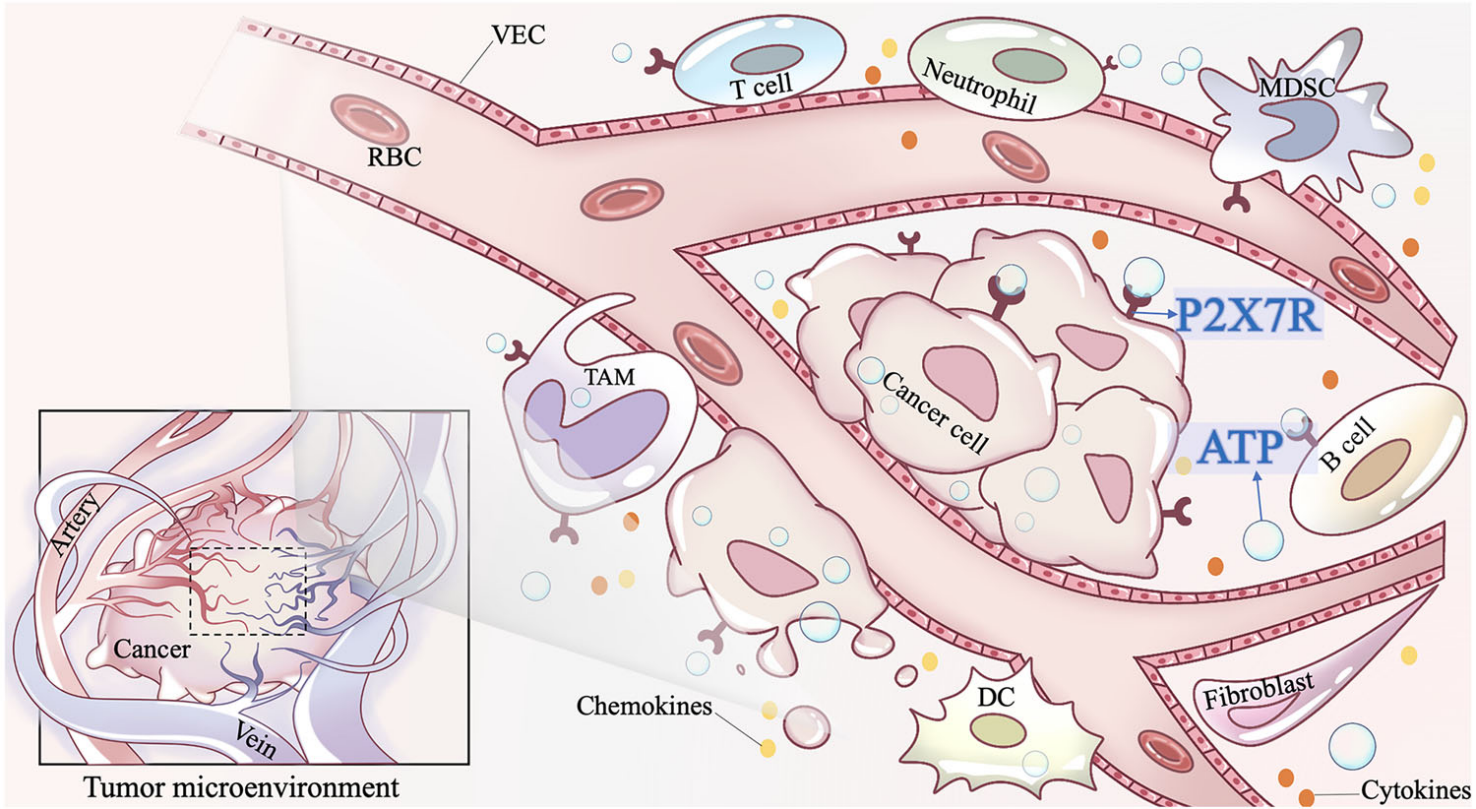
Schematic diagram of the tumor microenvironment. In the tumor microenvironment, P2X7R was expressed in almost all tumor cells and various immune cells. With the accumulation of extracellular ATP, P2X7R is activated and regulates tumor progression and antitumor immune responses. VEC, vascular endothelial cell; RBC, red blood cell; T cell, T lymphocyte; B cell, B lymphocyte; MDSC, myeloid‐derived suppressor cell; TAM, tumor‐associated macrophages; DC, dendritic cell.

In 1993, the concept of the TME was proposed.^82^ It was found that tumor cells can make full use of the various host resources to create a growth environment meeting their own growth needs, and thereby expand and transfer to the remote end.^83^ In the TME, in addition to tumor cells, there are also surrounding immune cells, fibroblasts, and vascular endothelial cells, as well as cellular stroma, microvessels, and various biomolecules infiltrating in nearby areas (Figure 2).^84^ Different cells interact with each other through direct contact or secreted cytokines,^85^ playing an important role in tumor progression.^13^ Therefore, modulating the immune function of TME may contribute to tumor therapy. In TME, in addition to common immune cells and immune molecules, purines and their derivatives have also been confirmed to be closely related to the immune regulation of tumors.^16^ In recent years, experimental evidence has demonstrated that ATP can be released into the TME through various mechanisms, thereby regulating signaling pathways in the TME via purinergic receptors and initiating a cascade of reactions. Among them, P2X7R, as a unique subtype of the ATP‐gated transmembrane ion channel receptor family, allows the passage of ions such as K^+^, Na^+^, and Ca^2+^, is activated, and participates in multiple functional activities.^86^

### 4.2 Accumulation of ATP

In general, ATP mainly accumulates inside the cell (3–10 mm), and it is difficult to estimate the ATP concentration outside the cell (usually a very low concentration in the nM range).^87^ ATP in physiological states is in a homeostatic balance, but the extracellular ATP (eATP) concentration is elevated under certain pathological conditions such as tumors.^63^, ^88^ The sources and mechanisms of eATP accumulation in the TME are diverse. First, ATP is passively released due to cellular damage caused by inflammation, hypoxia, chemotherapy drugs, and tissue destruction caused by tumor invasion.^89^ Second, active secretion of ATP from active or apoptotic cells via vesicular exocytosis, transporter, or membrane‐bound pathways also contributes to increased concentrations in TME.^90^, ^91^, ^92^ In the TME, eATP continuously accumulates, up to a level of several hundred micromoles,^88^ transducing active signals related to tumor cell metabolism and tumor immunity. The specific role of eATP in the TME depends on not only its concentration and the corresponding enzymes but also the specific purinergic receptor type and properties.^93^

### 4.3 The impact of purinergic receptors on tumors

#### 4.3.1 The impact of P1Rs on tumors

Under hypoxic conditions, ATP, ADP, and AMP continuously accumulate in the TME. Extracellular AMP is metabolized into adenosine under the action of CD73 and then activates adenosine receptors.^94^ In tumor cells, adenosine may simultaneously trigger both growth and inhibitory stimuli due to the differential expression of P1Rs and differential signal transduction.^46^ The role of A1R in tumors has been investigated, but its function remains unclear. Early studies have shown that A1R may promote tumor growth and angiogenesis in breast cancer, colorectal cancer, leukemia, and melanoma A375 cell lines.^95^, ^96^ However, subsequent studies have also demonstrated the potential anti‐tumor effects of A1R.

A2AR is highly expressed in certain tumors, such as head and neck squamous cell carcinoma and breast cancer. This high expression is significantly associated with increased tumor volume, advanced pathological grade, and diminished overall survival.^97^, ^98^, ^99^ The previous studies have shown that blocking A2AR in mice can enhance the immune response against tumors and inhibit tumor growth and metastasis.^100^ In the investigation of adenosine receptors’ role in cancer cells, A2BR and A2BR have emerged as two extensively studied receptors (Tables 2 and 3). The expression of A2BR is significantly higher in various types of tumors compared with normal tissues; thus, A2BR is considered as a potential biomarker.^101^ Tumor cells may exploit the characteristics of this receptor to facilitate their own progression. Studies have demonstrated that A2BR can increase vascular density by activating interleukin‐8 (IL‐8) and vascular endothelial growth factor (VEGF) promoters.^102^, ^103^ Conversely, inhibiting A2BR downregulation can impede tumor proliferation, migration, and invasion.^104^ A3R serves as the main receptor for adenosine, directly targeting tumor cells and exhibiting high expression in various malignant tumors of both epithelial and nonepithelial origin.^46^ Extensive research has been conducted on the role of A3R in different cancer cell types, revealing its ability to regulate proliferation, invasion, and apoptosis.^105^, ^106^, ^107^

**TABLE 2:** Clinical studies related to A2AR in ClinicalTrials.gov.

NCT number	Study title	Conditions	Phase	Study type	Status	First posted
NCT05501054	Phase 1b/2 trial of ipilimumab, nivolumab, and ciforadenant (adenosine A2a receptor antagonist) in first‐line advanced renal cell carcinoma.	Renal cell carcinoma	Phase 1, Phase 2	Interventional	Recruiting	August 15, 2022
NCT05177770	Study of SRF617 With AB928 (etrumadenent) and AB122 (zimberelimab) in patients with metastatic castration resistant prostate cancer	Metastatic castration‐resistant prostate cancer, prostate cancer	Phase 2	Interventional	Completed	January 5, 2022
NCT04969315	TT‐10 as a single agent in subjects with advanced selected solid tumors	Renal cell cancer, castrate resistant prostate cancer, non‐small cell lung cancer (NSCLC)	Phase 1, Phase 2	Interventional	Recruiting	July 20, 2021
NCT04895748	DFF332 as a single agent and in combination with everolimus & immuno‐oncology agents in advanced/relapsed renal cancer & other malignancies	Carcinoma, renal cell	Phase 1	Interventional	Recruiting	May 20, 2021
NCT04892875	A study of concurrent chemoradiation in combination with or without PD1 inhibitor AB122 adenosine 2a receptor/adenosine 2b receptor inhibitor AB928 therapies in locally advanced head and neck cancers	Head and neck cancer, squamous cell carcinoma of head and neck, oral cavity squamous cell carcinoma, oral cavity cancer, oropharynx cancer, oropharynx squamous cell carcinoma, larynx cancer, pharynx cancer, hypopharynx cancer, hypopharynx squamous cell carcinoma	Phase 1	Interventional	Not yet recruiting	May 19, 2021
NCT04660812	An open label study evaluating the efficacy and safety of etrumadenant (AB928) based treatment combinations in participants with metastatic colorectal cancer	Metastatic colorectal cancer	Phase 1, Phase 2	Interventional	Active, not recruiting	December 9, 2020
NCT04381832	Adenosine receptor antagonist combination therapy for metastatic castrate resistant prostate cancer	Prostatic neoplasms, castration‐resistant, androgen‐resistant prostatic neoplasms, castration resistant prostatic neoplasms, prostatic cancer, castration‐resistant	Phase 1, Phase 2	Interventional	Recruiting	May 11, 2020
NCT04266665	Effect of dexmedetomidine on brain homeostasis and neurocognitive outcome	Brain tumor, metabolic disturbance, inflammatory response, oxygen deficiency	Phase 4	Interventional	Completed	February 12, 2020
NCT04262856	Study to evaluate monotherapy and combination immunotherapies in participants with PD‐L1 positive non‐small cell lung cancer	NSCLC, nonsquamous NSCLC, squamous NSCLC, lung cancer	Phase 2	Interventional	Active, not recruiting	February 10, 2020
NCT04089553	An open‐label, phase II study of AZD4635 in patients with prostate cancer	Prostate cancer, metastatic castration‐resistant prostate cancer	Phase 2	Interventional	Completed	September 13, 2019
NCT03846310	A study to evaluate immunotherapy combinations in participants with lung cancer	NSCLC metastatic, NSCLC, nonsquamous non‐small cell neoplasm of lung, sensitizing EGFR gene mutation	Phase 1	Interventional	Active, not recruiting	February 19, 2019
NCT03720678	A study to evaluate immunotherapy combinations in participants with gastrointestinal malignancies	Gastroesophageal cancer, colorectal cancer	Phase 1	Interventional	Completed	October 25, 2018
NCT03719326	A study to evaluate safety/tolerability of immunotherapy combinations in participants with triple‐negative breast cancer or gynecologic malignancies	Triple‐negative breast cancer, ovarian cancer	Phase 1	Interventional	Completed	October 25, 2018
NCT03629756	A study to evaluate the safety and tolerability of immunotherapy combinations in participants with advanced malignancies	NSCLC, squamous cell carcinoma of the head and neck, breast cancer, colorectal cancer, melanoma, bladder cancer, ovarian cancer, endometrial cancer, Merkel cell carcinoma, gastroesophageal cancer, renal cell carcinoma, castration‐resistant prostate cancer	Phase 1	Interventional	Completed	August 14, 2018
NCT03549000	A phase I/Ib study of NZV930 alone and in combination with PDR001 and /or NIR178 in patients with advanced malignancies.	NSCLC, triple negative breast cancer, pancreatic ductal adenocarcinoma, colorectal cancer microsatellite stable, ovarian cancer, renal cell carcinoma, metastatic castration resistant prostate cancer	Phase 1	Interventional	Terminated	June 7, 2018
NCT03381274	Oleclumab (MEDI9447) epidermal growth factor receptor mutant (EGFRm) non‐small cell lung cancer novel combination study	Carcinoma, NSCLC	Phase 1, Phase 2	Interventional	Active, not recruiting	December 21, 2017
NCT03207867	A phase 2 study of NIR178 in combination with PDR001 in patients with solid tumors and non‐Hodgkin lymphoma	NSCLC, RCC, renal cell cancer, pancreatic cancer, urothelial cancer, head and neck cancer, diffused large B cell lymphoma, MSS, microsatellite stable colon cancer, triple negative breast cancer, melanoma, metastatic castration resistant prostate cancer	Phase 2	Interventional	Terminated	July 5, 2017
NCT02655822	Phase 1/1b study to evaluate the safety and tolerability of ciforadenant alone and in combination with atezolizumab in advanced cancers	Renal cell cancer, metastatic castration resistant prostate cancer	Phase 1	Interventional	Completed	January 14, 2016
NCT02403193	Trial of PBF‐509 and PDR001 in patients with advanced non‐small cell lung cancer	NSCLC	Phase 1	Interventional	Completed	March 31, 2015

**TABLE 3:** Clinical studies related to A2BR in ClinicalTrials.gov.

NCT number	Study title	Conditions	Phase	Study type	Status	First posted
NCT05272709	TT‐702 in patients with advanced solid tumors	Advanced solid tumors	Phase 1, Phase 2	Interventional	Recruiting	March 9, 2022
NCT05234307	PBF‐1129 and nivolumab for the treatment of recurrent or metastatic non‐small cell lung cancer	Metastatic lung non‐small cell carcinoma, recurrent lung non‐small cell carcinoma, stage IV lung cancer AJCC v8, stage IVA lung cancer AJCC v8, stage IVB lung cancer AJCC v8	Phase 1	Interventional	Recruiting	February 10, 2022
NCT05177770	Study of SRF617 with AB928 (etrumadenent) and AB122 (zimberelimab) in patients with metastatic castration resistant prostate cancer	Metastatic castration‐resistant prostate cancer, prostate cancer	Phase 2	Interventional	Completed	January 5, 2022
NCT04976660	TT‐4 as a single agent in subjects with advanced selected solid tumors	Colorectal cancer, gastric cancer, hepatocellular carcinoma, pancreatic cancer	Phase 1, Phase 2	Interventional	Not yet recruiting	July 26, 2021
NCT04892875	A study of concurrent chemoradiation in combination with or without PD1 inhibitor AB122 adenosine 2a receptor/adenosine 2b receptor inhibitor AB928 therapies in locally advanced head and neck cancers	Head and neck cancer, squamous cell carcinoma of head and neck, oral cavity squamous cell carcinoma, oral cavity cancer, oropharynx cancer, oropharynx squamous cell carcinoma, larynx cancer, pharynx cancer, hypopharynx cancer, hypopharynx squamous cell carcinoma	Phase 1	Interventional	Not yet recruiting	May 19, 2021
NCT04660812	An open label study evaluating the efficacy and safety of etrumadenant (AB928) based treatment combinations in participants with metastatic colorectal cancer	Metastatic colorectal cancer	Phase 1, Phase 2	Interventional	Active, not recruiting	December 9, 2020
NCT04381832	Adenosine receptor antagonist combination therapy for metastatic castrate resistant prostate cancer	Prostatic neoplasms, castration‐resistant, androgen‐resistant prostatic neoplasms, castration resistant prostatic neoplasms, prostatic cancer, castration‐resistant	Phase 1, Phase 2	Interventional	Recruiting	May 11, 2020
NCT04262856	Study to evaluate monotherapy and combination immunotherapies in participants with PD‐L1 positive non‐small cell lung cancer	NSCLC, nonsquamous NSCLC, squamous NSCLC, lung cancer	Phase 2	Interventional	Active, not recruiting	February 10, 2020
NCT03846310	A study to evaluate immunotherapy combinations in participants with lung cancer	NSCLC metastatic, NSCLC, nonsquamous non‐small cell neoplasm of lung, sensitizing EGFR gene mutation	Phase 1	Interventional	Active, not recruiting	February 19, 2019
NCT03720678	A study to evaluate immunotherapy combinations in participants with gastrointestinal malignancies	Gastroesophageal cancer, colorectal cancer	Phase 1	Interventional	Completed	October 25, 2018
NCT03719326	A study to evaluate safety/tolerability of immunotherapy combinations in participants with triple‐negative breast cancer or gynecologic malignancies	Triple‐negative breast cancer, ovarian cancer	Phase 1	Interventional	Completed	October 25, 2018
NCT03629756	A study to evaluate the safety and tolerability of immunotherapy combinations in participants with advanced malignancies	NSCLC, squamous cell carcinoma of the head and neck, breast cancer, colorectal cancer, melanoma, bladder cancer, ovarian cancer, endometrial cancer, Merkel cell carcinoma, gastroesophageal cancer, renal cell carcinoma, castration‐resistant prostate cancer	Phase 1	Interventional	Completed	August 14, 2018
NCT03274479	PBF‐1129 in patients with NSCLC	Locally advanced or metastatic	Phase 1	Interventional	Active, not recruiting	September 7, 2017

#### 4.3.2 The effect of P2XRs on tumors

Currently, due to the emphasis on P2X7R in the study of P2XRs‐mediated purinergic signaling, further evidence is required to ascertain the involvement of other receptors in tumor progression and elucidate their respective roles. The role of P2X7R in tumors will be discussed in detail later, while other P2XRs will be introduced here. As a receptor related to human hematopoiesis, P2X1R is upregulated in leukemia and correlates with the malignant phenotype of the disease.^108^ The concurrent administration of P2X1R or P2X7R inhibitors in combination with chemotherapy drugs has the potential to effectively inhibit the proliferation of Jurkat acute T lymphocytic leukemia cells.^109^ P2X2R and P2X3R may have an influence on pain‐related behaviors in early‐stage tumors.^110^ Furthermore, high expression of P2X3R is associated with decreased recurrence‐free survival.^111^ In hepatocellular carcinoma, the upregulation of P2X4R exacerbates inflammatory responses.^112^, ^113^ Silencing P2X4R through the BDNF/TrkB/ATF4 signaling pathway can inhibit the growth of human glioblastoma multiforme cells.^114^ Positive expression of P2X4R and P2X5R has been detected in prostate cancer and leukemia.^108^, ^115^ The expression characteristics of P2X5R may have prognostic and predictive value in colon cancer.^116^ Additionally, P2X6R plays an important role in the development of human renal cell carcinoma. The ATP‐P2X6R can regulate the p‐extracellular signal‐regulated kinase 1/2 (p‐ERK1/2) /matrix metalloproteinase 9 (MMP9) signaling pathway, thereby promoting the migration and invasion of renal cell carcinoma cells.^117^

#### 4.3.3 The impact of P2YRs on tumors

With the continuous advancement of the related detection techniques, in vivo and in vitro animal models, our understanding of the role of P2YRs in cancer occurrence and development has been continuously deepened and translated into clinical treatment. Studies have found that although P2Y1R is expressed in various human tissues, its distribution is most prominent within the prostate.^118^ When P2Y1R is activated, it triggers cellular apoptosis through the Capase3/7 and reactive oxygen species (ROS) signaling pathways, suggesting that P2Y1R agonists have the potential utility as therapeutic agents for prostate cancer.^119^ In addition to P2Y1R, the involvement of P2Y2R is also pivotal in the pathogenesis and progression of prostate cancer. P2Y2R exerts its influence on the invasion and metastasis of prostate cancer cells through modulation of the EGFR–ERK1/2 pathway and associated targets, including IL‐8, E‐cadherin, Snail, and Claudin‐1.^120^ Furthermore, P2Y2R can induce a DNA damage response and promote hepatocyte proliferation, thereby facilitating the development of liver cancer in mice.^121^ P2Y4R has been shown to regulate the progression of human breast cancer cells MCF‐7 through the PKC/MAPKs and PKC/Src pathways,^122^ as well as participate in the differentiation and cell death process of human neuroblastoma SH‐SY5Y cells.^123^ In intestinal tumors, sustained activation of P2Y6R may impede the process of cellular apoptosis and promote the resistance to chemotherapy.^124^ Additionally, signaling through the Gαq/Ca^2+^/PKCα and Gα13/ROCK pathways stimulates cell migration.^125^ In breast cancer, the overexpression of P2Y6R triggers cell migration and invasion into the extracellular matrix (ECM),^126^ whereas the activation of P2Y11R inhibits migration through cAMP signaling and normalizes tumor‐derived endothelial cells.^127^ In lung cancer tissue, there is a relative downregulation of P2Y12R, P2Y13R, and P2Y14R, which typically indicates a favorable prognosis.^128^

## 5 P2X7R IN TME

The long‐term observation results have provided evidence for the presence of elevated concentrations of eATP in TME, which can induce specific cytotoxic effects on antitumor immune responses.^86^ The unique recognition of eATP as a natural agonist by P2XRs has garnered significant attention, highlighting their prominence among purinergic receptors. The exceptional specificity of this ligand not only enhances the characterization activity of P2XRs but also facilitates the development of superior antagonists,^129^ rendering P2XRs an ideal target for antitumor drug discovery. Furthermore, the immunological effects of eATP are mediated by the P2X7R subtype in P2XRs in most cases.^130^ P2X7R is not only widely expressed in tumor cells, but also the most prevalent and highly expressed subtype in immune cells.^129^ Based on pharmacological evidence, P2X7 receptors are expressed by almost all innate and adaptive immune cells and exhibit a more pronounced involvement in immune responses and inflammatory reactions compared with other subtypes.^131^ In addition, the distinctiveness of P2X7R is also evident in its capacity to activate cation‐selective ion channels and nonselective pores. Given that TME has a sufficient level of eATP for P2X7R activation,^88^ P2X7R may express completely opposite functions (i.e., triggering cell death or supporting growth) depending on the activation level and cell type. Therefore, this review aims to provide a comprehensive overview of the role played by P2X7R in tumorigenesis.

### 5.1 Expression of P2X7R in TME

Due to its low affinity for ATP, P2X7R differs slightly from other receptors,^132^ while it is widely distributed in the vast majority of tissue cells and plays a role in biological regulation, such as immune cells, nerve cells, and tumor cells.^133^ Accumulating evidence has shown that compared with normal tissues, P2X7R is highly expressed in a variety of tumors, such as acute myeloid leukemia,^134^ breast cancer,^135^ prostate cancer,^136^ thyroid cancer,^137^ pancreatic ductal malignant adenoma,^138^ while its expression is lower in some tumors.^139^ Some researchers claimed that this may be related to the embryological context of the tumor,^139^ that is, in tumor cells derived from endoderm, P2X7R is present at higher or equal levels to normal cells, whereas in tumor cells derived from ectoderm (skin and breast), urogenital sinus (bladder and ectocervix), and distal pararenal ducts (endocervical canal and endometrium), P2X7R is present at lower to normal cells. In addition, there is an association between the expression of P2X7R and disease severity and prognosis.^140^, ^141^ For example, Adinolfi et al.^140^ examined the P2X7R expression in chronic B‐lymphocytic leukemia and found that the expression of P2X7R in patients at progression was significantly higher than that at quiescence. Alternatively, the expression of P2X7R may change with the stage progression of the tumor. Specifically, in prostate cancer, the distribution of P2X7R is mainly concentrated in the nucleus, then extends to the cytoplasm, and finally accumulates in the apical membrane of epithelial cells.^142^ The expression of P2X7R can further regulate the release of ATP from tumor cells and immune cells in the TME, which in turn amplifies P2X7R signaling or acts on other purinoceptor subtypes to regulate tumor growth and antitumor immune responses.^143^, ^144^

### 5.2 The role of P2X7R in tumor growth and proliferation

**FIGURE 3:**
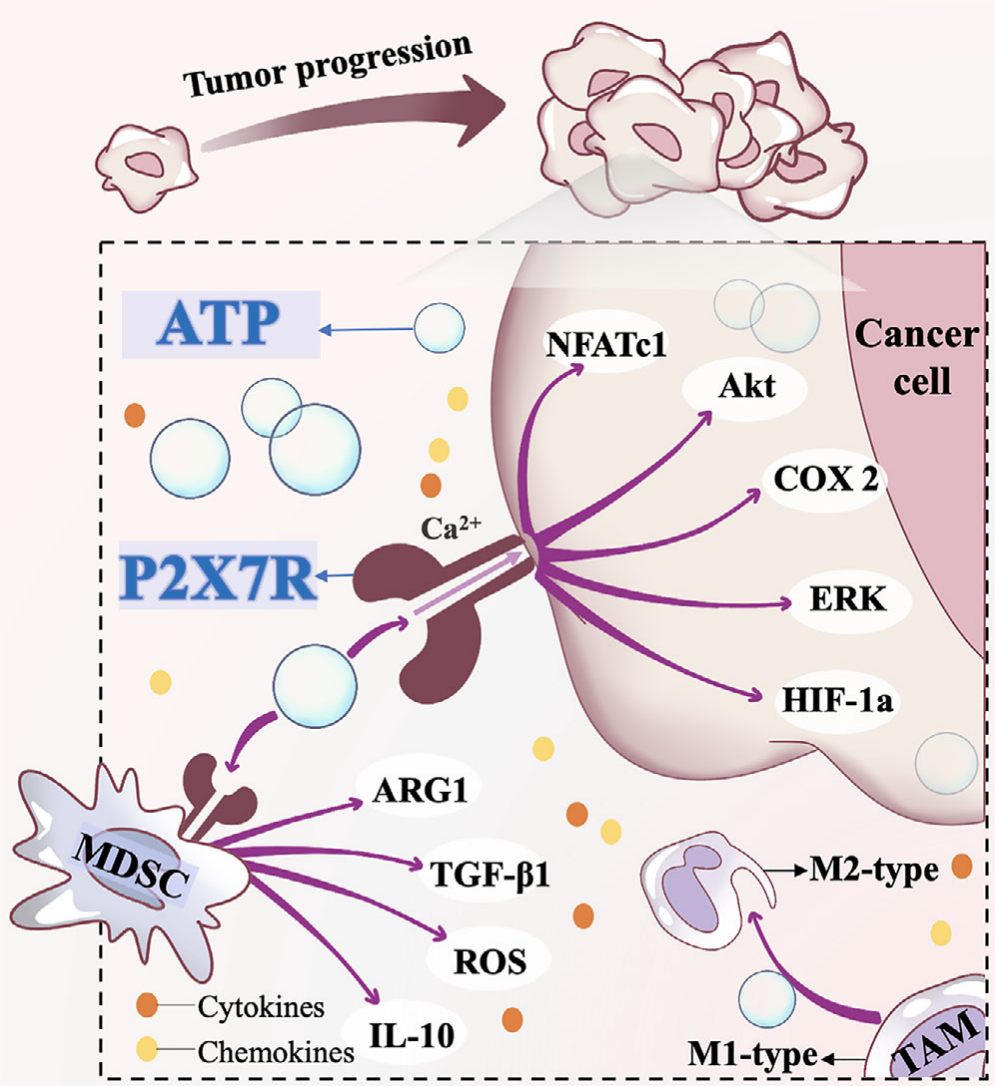
The potential mechanism of P2X7R overexpression causing tumor progression. High expression of P2X7R can alter the infiltration of immune cells in TME, creating an immunosuppressive microenvironment. At the same time, P2X7R also activates intracellular growth promotion pathways such as NFATc1, ERK, Akt, and HIF‐1a. NFATc1: nuclear factor‐activated T cell 1; ERK, extracellular regulated protein kinases; Akt, protein kinase B; HIF‐1a, hypoxia‐inducible factor‐1α; COX<: cyclooxygenase‐2; MDSC, myeloid‐derived suppressor cell; TAM, tumor‐associated macrophages; ARG1, arginase 1; TGF‐β1, transforming growth factor‐β1; ROS, reactive oxygen species; IL‐10, interleukin‐10.

The initiation and growth of tumors are regulated by various carcinogenic factors, and P2X7R is one of the important carcinogenic factors (Figure 3). As a potent stimulant of inflammation and immunity, P2X7R confers a strong growth‐promoting advantage.^145^ It not only enhances the index of tumor cell division, increases the proliferation, decreases the apoptosis, but also has high levels of the activating transcription factor nuclear factor‐activated T cell 1 (NFATc1).^146^, ^147^ In vivo studies have found that compared with normal control cells, human embryonic kidney cells expressing P2X7R have greater tumorigenicity and a more obvious undifferentiated carcinoma phenotype.^146^ The expression and function of P2X7R alter immune cell infiltration and eATP content of the TME, thus affecting tumor growth.^143^ P2X7R can mediate the release of ATP to the extracellular space by tumor cells to cope with the acidic TME of local hypoxia and interstitial hypertension caused by the high metabolism of tumors.^147^ Meanwhile, activated P2X7R can increase the Ca^2+^ concentration and mitochondrial thickness in tumor cells, promote the energy reserves of cells to maintain tumor growth through oxidation, glycolysis, and other pathways,^148^ and inhibit cell apoptosis.^146^, ^149^ In addition to its effects on energy metabolism, P2X7R activates several key intracellular growth‐promoting pathways, including extracellular regulated protein kinases (ERK),^150^ protein kinase B (Akt), and hypoxia‐inducible factor‐1α (HIF‐1a).^141^, ^151^

### 5.3 The role of P2X7R in tumor metastasis and invasion

In addition to promoting tumor proliferation, P2X7R also enhances tumor cell extension, migration, and invasiveness by regulating targets (such as E‐cadherin, ERK1/2, cyclooxygenase protein 2) and signaling pathways.^142^, ^152^ First, actin filaments are important components of the cytoskeleton, and ATP‐P2X7 signaling drives its dynamic remodeling and serves as a structural basis and source of motility for morphological changes and migratory movements of tumor cells.^153^ Second, the ECM provides a supporting framework for tissue construction and also serves as a histological barrier to regulate tumor cell metastasis. Jelass et al.^154^ demonstrated that in highly aggressive human breast cancer cell lines, P2X7R can degrade EMC and enhance the migration and aggressiveness of tumor cells by 35 and 150%, respectively. P2X7R stimulation can induce CD44 shedding and affect the mutual adhesion between tumor cells and EMC,^155^ or evoke the release of MMPs, cysteine cathepsins^156^, ^157^, ^158^ from monocytes and macrophages^159^, ^160^ and disrupt protein components in the ECM.^161^ It is important to note that the high expression of P2X7R may produce different outcomes in different tumors. For instance, in basal cell carcinoma and squamous cell carcinoma, the expression of P2X7R on tumor cells with high invasiveness is paradoxically reduced.^162^

## 6 THE CORRELATION BETWEEN P2X7R AND TUMOR IMMUNITY

Researchers are trying to solve the challenging health problem of cancer in a variety of ways. In addition to the traditional surgical procedures, chemotherapeutic drugs and radiotherapy, the discovery of many specific targets holds promise for further precise targeting and reducing damage to normal tissue. Besides, the 2018 Nobel Prize in Physiology has been awarded to the field of tumor immunotherapy, which has pioneered a new direction in the treatment of cancer and brought light to many patients with advanced diseases. However, immunotherapy is not effective for all tumors, and such tumors that are insensitive to immunotherapy are called cold tumors.^163^ The core focus of future drug development lies in activating immune function for tumors lacking immune infiltration through alternative pathways of antigen presentation, and the distinctive properties of P2X7R distinctive it a potential therapeutic target.

### 6.1 Immune features and challenges of TME

Immunotherapy can activate the body's own immune defense system and is widely used in the treatment of a variety of malignancies. However, the therapeutic effect of immunotherapy for most patients is not significant.^164^ It is increasingly recognized that a positive response to immunotherapy often depends on the interaction of tumor cells with immune regulation within TME. In general, the activation of the immune system, expansion of effector cells, recognition of target antigens, and destruction of tumor cells are key steps in the development of curative effects.^165^ However, the phenomenon of tumor refractoriness and relapse suggests that the TME has diversity and heterogeneity,^166^ which may block the immunotherapy effect through compensatory mechanisms and dynamic evolution. As a result, drug resistance in the body can be caused, and tumor escape and disease progression can be promoted.^167^ The TME of refractory tumors is generally classified into immune‐privileged and inflammatory types. In the inflammatory TME, tumor cells coexist with enriched active T cells and myeloid cells and have chemokine, type I interferon (IFN) signaling expression.^168^ In contrast, only a small number of immune cells or inhibitory subpopulations, such as regulatory T cells (Tregs), myeloid‐derived suppressor cells (MDSCs), and tumor‐associated macrophages (TAMs) are present in the immune‐privileged TME (i.e., cold tumor), whereas effector immune cells are only distributed in the peripheral matrix and cannot infiltrate efficiently into the TME, so that their tumor suppressive functions cannot be exerted effectively.^169^ The close association between P2X7R and TME suggests that leveraging this connection can be advantageous in addressing the current treatment dilemma.

### 6.2 P2X7R modulates tumor immune escape and drug resistance

Tumor immune escape refers to the process through which tumor cells evade recognition and attack by the immune system by modifying themselves or affecting the surrounding immune environment. For instance, tumors can evade immune responses by utilizing the inhibitory immune checkpoint programmed cell death‐1 (PD‐1)/programmed cell death ligand‐1 (PD‐L1) pathway.^170^ Moreover, the process of epithelial–mesenchymal transition (EMT) significantly enhances the cancer cell chemoresistance by inducing a phenotypic switch from stationary epithelial cells to motile mesenchymal cells, thereby altering cellular adhesion and ECM composition.^171^, ^172^, ^173^ P2X7R can induce the upregulation of EMT markers in tumor cells through its mediation of MMP 2, ERK1/2, PI3K/Akt, and other signaling pathways, thus driving the development of drug resistance.^174^, ^175^, ^176^ Moreover, P2X7R modulates the recognition and clearance ability of immune cells toward tumor cells by influencing the expression of surface antigens on tumor cells. For example, the activation of P2X7R can potentially evade the immune response by inhibiting the levels of MHC I in tumor cells and suppressing MHC I‐related antigen presentation in macrophages.^177^ The systemic blockade of P2X7R triggers immunogenic cell death mechanisms within cancer cells. As a result, the reduced tumor growth and activation of antitumor responses can be caused, and the protumor effects of inflammatory cytokine IL‐1β are simultaneously reduced.^178^

### 6.3 P2X7R changes the tumor's immune microenvironment

In addition to acting on tumor cells, P2X7R also exerts its influence on the TME as a whole and is a key determinant of tumor–host interactions. The P2X7R has the ability to regulate immune cell infiltration, extracellular nucleotidase expression, and extracellular ATP levels within TME.^143^ For example, the over‐expression of P2X7R in MDSCs can trigger the expression and/or release of immunosuppressive factors, such as IL‐10, arginase 1 (ARG1), transforming growth factor‐β1 (TGF‐β1) and ROS, creating an immunosuppressive TME. Therefore, P2X7R not only promotes tumor growth but also antagonizes the effects of immunotherapy.^179^ Recent studies have confirmed^180^ that P2X7R can promote M2‐type polarization of TAMs in the TME, which in turn exerts Pro angiogenic, tissue remodeling and promotes tumor growth and metastasis.^181^ Additionally, to allow tumor cells to draw more oxygen and nutrients, angiogenic factors in the TME activate an “ angiogenic switch ” and induce the establishment of neovasculature.^182^ Among them, the activation of P2X7R generates molecular building blocks required to support cell proliferation, thus driving tumor neovascularization.^133^, ^183^ Amoroso et al.^141^ confirmed that in neuroblastoma, P2X7R can promote the secretion of VEGF through the PI3K/GSK3β/MYCN/HIF1α axis, thus promoting angiogenesis.

## 7 IMMUNOTHERAPY AGAINST P2X7R

Research on antitumor therapy has focused on the tumor itself, although surgery or highly toxic therapies can cause unavoidable substantial harm to the normal tissue cells of the body. With further study of the complex information exchange network in TME, there is an opportunity to halt tumor progression through more gentle and efficient immunological interventions. Immunotherapy is a new therapeutic switch that activates antitumor T‐cell immunity to improve the clinical outcomes of patients.^184^ The above results suggest that targeting P2X7R to alter metabolic processes of tumor cells or other biomolecules in the TME, such as through the P2X7R‐NLRP3 pathway, is a highly potential approach for tumor immune prevention and treatment. P2X7R‐NLRP3 activation can promote immune infiltration, but the excessive inflammatory environment is also conducive to maintaining the survival and proliferation of tumor cells.^185^ Indeed, the initiation of the immune system depends on the tumor immunity cycle, including the release and presentation of tumor antigens, activation and migration of T cells, infiltration and recognition of tumor tissues.^186^ Targeting P2X7R can produce a series of chain reactions in TME, in which abnormalities in any link may contribute to immune escape. Thus, precise regulation of P2X7R is key to the development of therapeutic efficacy.

### 7.1 Bidirectional nature of P2X7R

**FIGURE 4:**
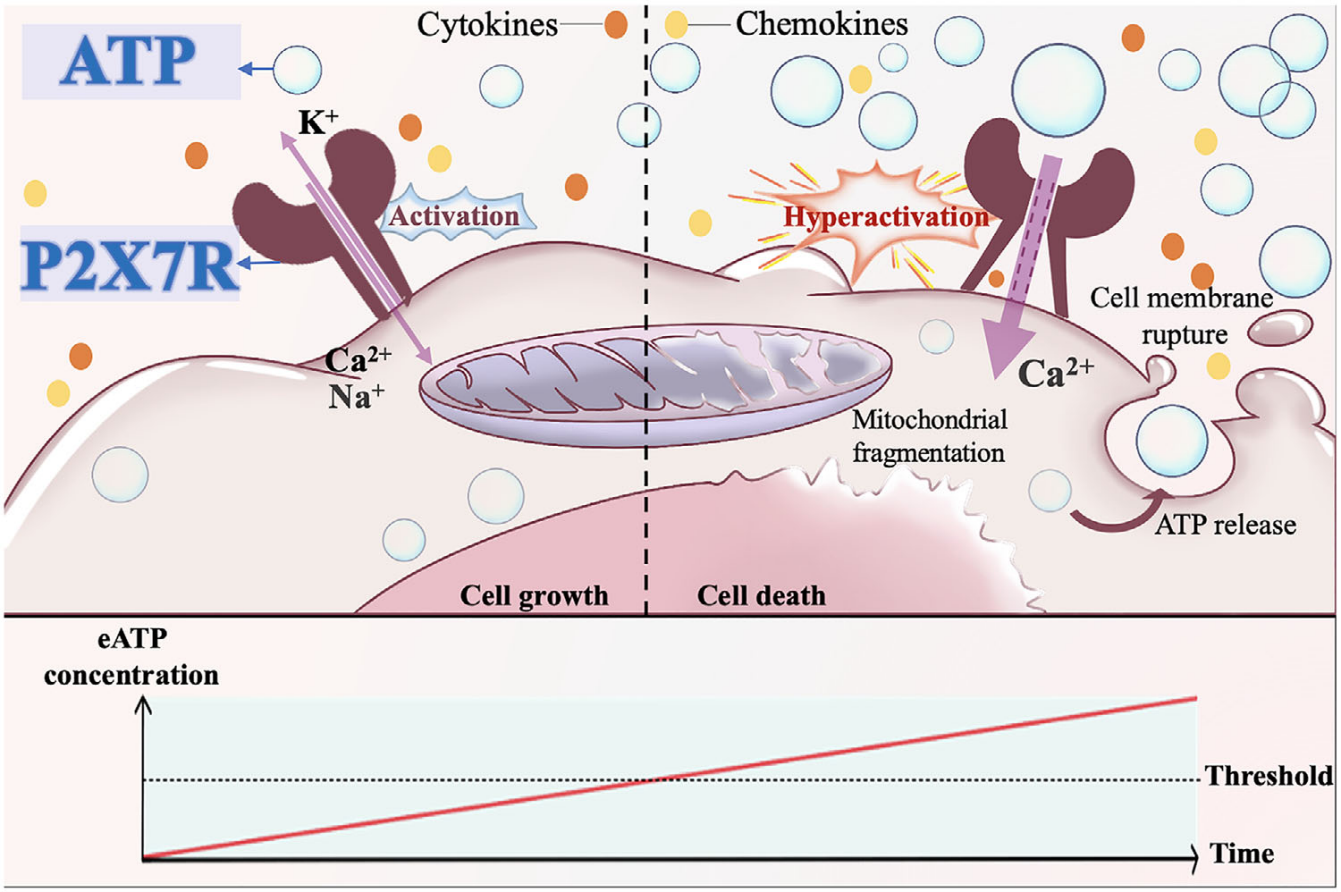
The bidirectional nature of P2X7R. In TME, eATP stimulation promotes P2X7R to open channels that allow small cations to permeate, which is beneficial for tumor cell growth. As the concentration of eATP increases, the action time is prolonged. When the threshold value is exceeded, it will drive the nonselective macropore opening of P2X7R, the infiltration of large ions such as inflammatory cytokines, and lead to tumor cell lysis and death.

As a constantly changing dynamic environment, the TME can both provide a protective antitumor immune response and create an immunosuppressive environment that promotes tumor progression (Figure 4). Tumors affect other components of the TME by releasing cell signaling molecules, promoting angiogenesis, and inducing immune tolerance. Conversely, immune cells in the microenvironment can also regulate tumor cell metabolism and motility. For example, the expression of tumor P2X7R can promote the growth, invasion, and metastasis of tumor cells, while the expression of host P2X7R can trigger a tumor immune response through inflammatory cell infiltration, such as the release of IL‐1β and other inflammatory factors by dendritic cells (DCs) and macrophages, and inhibit tumor progression.^187^, ^188^ eATP stimulation enables cGAMP to enter host immune cells via P2X7R, which promotes innate immune sensing of tumor cells and type I IFN production.^189^ Correspondingly, the deficiency of host P2X7R leads to the decrease of CD8 and Teff cells and the increase of immunosuppressive Tregs, contributing to cancer progression.^143^ In the absence of host P2X7R, the increased expression of A2AR receptors promotes tumor growth by inducing immune suppression and neovascularization.^190^ Moreover, under the transient stimulation of low concentration of eATP and its analogs, P2X7R opens the channel allowing small cations to permeate, promoting tumor cell proliferation and immune response. However, a high concentration of eATP or continuous stimulation of repeated action may drive the nonselective macropore opening, and the infiltration of large ions such as inflammatory cytokines can cause irreversible damage, and eventually lead to tumor cell lysis and death.^191^, ^192^

### 7.2 Tumor P2X7R inhibition

The growth and invasion of most tumors are closely associated with overexpression of P2X7R,^16^ and P2X7R is regarded as a potential therapeutic target for cancer due to its unique immune triggering effect.^193^ P2X7 antagonists are able to inhibit the overall progression of breast cancer,^194^ colon cancer,^146^ mesothelioma,^195^ osteosarcoma,^196^ melanoma^147^ and other tumors. Amoroso et al.^141^ found that P2X7 antagonists can cause the downregulation of the Akt/HIF1α axis, leading to a decrease in VEGF content and reduced vascularization in the TME. Currently, many pharmaceutical companies have synthesized small molecule (drug‐like) P2X7R inhibitors and used them in phase I and phase II clinical studies for some chronic inflammatory diseases,^197^, ^198^, ^199^ and their main side effects include gastrointestinal discomfort (diarrhea and nausea), dizziness, and headache.^200^ However, the effects of P2X7R in different tumors are specific. Hofman et al.^201^ found that P2X7R antagonists can inhibit intestinal inflammation in a mouse model of colon cancer, but paradoxically promotes the proliferation of tumor cells. This may be related to an immunosuppressive microenvironment prone to tumor development established by upregulation of TGF‐β1, accumulation of Tregs, and reduced STAT3 phosphorylation. Therefore, the use of P2X7R inhibitors remains to be further studied.

### 7.3 Tumor P2X7R hyperactivation

In addition, compared with low‐level P2X7R stimulation in promoting cell survival and proliferation, the overstimulation of P2X7R tends to lead to opposite outcomes.^140^ P2X7R belongs to ligand‐gated cation channels; upon stimulation, P2X7R can mediate the selective passage of Na^+^, K^+^, and Ca^2+^ .^202^ In addition to the canonical ion channel, P2X7R can also generate a nonselective pore permeable to aqueous solutes of MW up to 900 Da.^31^ Overactivated P2X7R further drives the release of proinflammatory cytokines through different mechanisms, such as exocytosis via secretory lysosomes, microvesicles shedding from plasma membrane, the release of exosomes, and passive efflux across a leaky plasma membrane during pyroptotic cell death.^203^ Overactivation of host P2X7R by ATP also has a killing effect on tumor cells.^16^, ^204^ Strong stimulation by ATP will lead to massive calcium overload of mitochondria, extensive fragmentation of the mitochondrial network, and cell death, and P2X7R will be transformed from a growth‐promoting to a proapoptotic receptor.^19^ Tamajusuku et al.^205^ found that high concentrations of ATP or P2X7 agonists can induce membrane penetration and rupture of glioma cells, resulting in the necrosis. Meanwhile, high concentrations of ATP can act on tumor endothelial cells as an endogenous stabilizing factor to promote vascular normalization.^127^

### 7.4 Host P2X7R activation

Besides inhibiting tumor P2X7R expression, host P2X7R activation by ATP also has a killing effect on tumor cells.^206^ P2X7R is expressed in almost all innate and adaptive immune cells^131^ and is proven to trigger immune responses leading to cells undergoing necrosis, pyroptosis, or apoptosis.^207^, ^208^ The activation of P2X7R can enhance the cytotoxicity of immune cells and promote immune recognition and elimination of tumor cells. Upon P2X7R induction, the NLRP3/caspase inflammasome signaling complex is activated and drives proinflammatory cytokine secretion, resulting in significantly increased expression of Caspase‐1, IL‐1β, and IL‐18 and pyroptosis.^209^ The occurrence of pyroptosis contributes to an improved TME and improved efficacy of immunotherapy.^210^ Under the action of anticancer drugs, dying cancer cells release a large amount of intracellular ATP,^211^ which is recognized by macrophages and DCs in TME and initiates the anticancer activity of T lymphocytes in a P2X7R–NLRP3–ASC–Casp‐1–IL‐1β‐dependent manner. The increase of T cell infiltration in the tumor is one of the key windows to promote the efficacy of cancer immunotherapy.^206^ To note, Lecciso et al.^212^ found that this local ATP effect may drive indoleamine 2,3‐dioxygenase‐1 upregulation in DCs in a P2X7R‐dependent manner and allow Tregs to produce an immunosuppressive microenvironment.

### 7.5 The clinical application prospects of P2X7R in tumor therapy

The presence of tumors poses a significant global health challenge, greatly impacting the quality of life and survival rates of patients. Given the pivotal role played by P2X7R in tumor metabolism, researchers are actively exploring therapeutic approaches centered around this target. Currently, pharmaceutical companies have synthesized small molecule agents targeting P2X7R, and some of these drugs have successfully progressed into clinical trials^213^ (Table 4). In a recent phase 1 clinical trial of a topical ointment containing anti‐P2X7 antibody BIL010t, 65% of basal cell carcinoma patients exhibited a reduction in lesion area after 28 days of treatment, and the treatment was well‐tolerated.^214^ Australia has initiated safety and immunogenicity evaluations of an anti‐P2X7 vaccine (BIL06v) for patients with advanced solid tumors.^178^ Several P2X7R antagonists that are still in development have demonstrated potential anticancer effects by inhibiting cell proliferation, invasion, and migration, including oxidized ATP, A740003, A438079, GSK1482160, JNJ‐54175446, and JNJ‐55308942.^215^ Additionally, natural compounds derived from nature have demonstrated the ability to inhibit P2X7R function and possess potential therapeutic value, such as baicalein, teniposide, and emodin.^216^, ^217^, ^218^ It is worth noting that due to uncertainties in their efficacy, off‐target effects, and adverse reactions, further research and extensive data evaluation of the type, method, and dosage of administration are required to maximize therapeutic efficacy for these molecules and compounds.

**TABLE 4:** Clinical studies related to P2X7R in ClinicalTrials.gov.

NCT number	Study title	Conditions	Phase	Study type	Status	First posted
NCT04116606	Antidepressant trial with P2X7 antagonist JNJ‐54175446	Major depressive disorder	Phase 2	Interventional	Active, not recruiting	October 4, 2019
NCT03918616	P2X7 receptor, inflammation and neurodegenerative diseases	Neuro‐degenerative disease	–	Observational	Completed	April 17, 2019
NCT00293189	Gene‐polymorphies in the P2X7 gene in patients with osteoporotic fractures	Hip fracture	–	Observational	Unknown	February 17, 2006
NCT02082821	A P2X7R single nucleotide mutation promotes chronic allograft vasculopathy	Cardiac allograft vasculopathy	–	Observational	Completed	March 10, 2014
NCT03437590	A positron emission tomography (PET) study to investigate P2X7 receptor occupancy by JNJ‐55308942 using [18F]‐JNJ‐64413739	Healthy	Phase 1	Interventional	Completed	February 19, 2018
NCT02587819	Investigation of the safety and tolerability of BSCT (Anti‐nf‐P2X7) 10% ointment	Basal cell carcinoma	Phase 1	Interventional	Completed	October 27, 2015
NCT03088644	A study to investigate P2X7 receptor occupancy by JNJ‐54175446 with the newly developed P2X7 receptor positron emission tomography (PET) tracer 18F‐JNJ‐64413739	Healthy	Phase 1	Interventional	Completed	March 23, 2017
NCT05753995	Immuno‐PET‐glioma study, a proof‐of‐principle imaging study	Glioblastoma	Not applicable	Interventional	Not yet recruiting	March 3, 2023
NCT00471120	Feasibility study: accuracy of biomarker in detection of endometrial cancer	Uterine cancer, endometrial cancer	Not applicable	Interventional	Terminated	May 9, 2007
NCT00628095	Study of CE‐224,535 a twice daily pill to control rheumatoid arthritis in patients who have not totally improved with methotrexate	Arthritis, rheumatoid	Phase 2	Interventional	Completed	March 4, 2008
NCT02293811	Decoding of the expression of tumor suppressor P2RX7 in inflammatory and malignant colonic mucosa	Crohn disease‐associated colorectal adenocarcinoma	Not applicable	Interventional	Unknown	November 18, 2014

The management of cancer pain should not be limited to the tumor itself, but also consider the pain caused by the underlying pathology. Research data indicate that nearly 50% of cancer patients experience pain, particularly those with advanced tumors, the incidence of pain in cancer patients is close to 50%, especially for patients with advanced tumors, where persistent pain can significantly affect their quality of life and emotional well‐being.^219^, ^220^ Currently, the three‐step analgesic plan is widely used for cancer pain management, which involves nonsteroidal anti‐inflammatory drugs, mild opioids, and potent opioids based on the severity of pain along with other physical or chemical therapies. However, research has also revealed that some patients exhibit poor efficacy, intolerance, or severe adverse reactions to this treatment plan.^221^, ^222^ Studies have found that P2X7R can mediate inflammatory reactions by releasing proinflammatory cytokines such as tumor necrosis factor‐α and IL‐1β, activating the central downstream emmetropic system to enhance pain transduction and transmission.^223^ The activation of the central downstream emmetropic system by P2X7R may contribute to the exacerbation of cancer pain and comorbid depression. Therefore, targeting P2X7R may be a valuable therapeutic approach for managing cancer pain and secondary depression.^224^

## 8 SUMMARY AND OUTLOOK

The proposal of “purinoceptor” as a ubiquitous intercellular communication system was made over 40 years ago. Since then, purinergic signaling has achieved significant progress in both physiology and pathology research, establishing itself as an indispensable cell‐to‐cell communication mechanism. The changes of ATP and adenosine concentrations hold distinct biological significance, while the expression of different P1 and P2 receptor subtypes brings about diverse clinical manifestations and therapeutic potential. To fully utilize their benefits, a comprehensive understanding of purinergic signaling and purinergic receptors is essential. Purinergic signaling and purinergic receptors play crucial roles in various biological processes, encompassing cell proliferation, differentiation, metabolism, motility, apoptosis, and intercellular signal transduction. In the majority of human cancers, alterations occur in the activity of purinergic receptors leading to either hyperactivity or inactivity, which often correlates with distinct clinical outcomes. Consequently, these receptors are regarded as promising therapeutic targets for cancer treatment.

Tumors are neoplasms that arise from the abnormal proliferation and differentiation of body cells under various factors. In contrast to normal cells, tumor cells grow exhibit uncontrolled growth, and disrupt normal physiological functions. The immune function of most malignant tumor patients is variably compromised, rendering the body's immune system incapable of initiating regular immune programs to suppress tumor progression. A wealth of clinical evidence has shown that the intricacy of cancer necessitates a treatment approach that extends beyond the use of external agents to eliminate tumor cells. More importantly, it is imperative to activate the body's immune function to actively target and attack affected tissue for optimal results. In fact, tumors can evade immune surveillance through various mechanisms, thereby enabling their survival and proliferation within the host. In recent decades, the rapid development of tumor immunotherapy has underscored the critical interplay between the human immune system and cancer. However, only a few late‐stage cancer patients have been able to derive long‐term survival benefits from this approach. How to make the application of immunotherapy more extensive and efficient is still an unresolved problem.^225^ To broaden the scope of antitumor treatment, it is imperative to identify and develop novel therapeutic targets and innovative molecules/strategies.

Undoubtedly, P2X7R is actively involved in tumor progression and regulates cell proliferation or apoptosis. Therefore, P2X7R can be used as a potential target for tumor therapy. At present, based on the characteristics of P2X7R in the TME, the development of safe and effective immunotherapy is one of the research hot spots of antitumor technology.^199^ The role of P2X7R depends on a number of factors, including the concentration and timing of agonists in TME, cell type, and host–tumor interaction.^226^ Therefore, the development of immune drugs requires a special focus on the impact of P2X7R basal expression. In addition, the distribution and context of immune cells differ in the TME of different tumors, which leads to significant differences in the efficacy of antitumor immunotherapy. In the future, how to regulate antigen presentation in the TME lacking immune infiltration is also the key to the development of immune drugs, and P2X7R is a potential target because of its special bidirectionality. How to accurately balance the efficacy of immunotherapy and regulate tumor growth by regulating P2X7R is one of the key concerns in the future. In summary, the TME has a complex dynamic environment and individual characteristics, it is necessary to deeply understand the position, state, and interaction of various cells in TME, especially the biology and function of nonmalignant components. Besides, the action law, mechanism and key links of P2X7R under physiological and pathological conditions should be accurately grasped, and suitable population and combined immunotherapy strategies should be optimized.

## AUTHOR CONTRIBUTIONS

Shiyun Tang, Jianyuan Tang, and Nianzhi Chen conceived the study and revised the manuscript. Yanling Ai, Hengyi Wang, and Lu Liu wrote the paper. Yulin Qi collecting references and writing. All authors have approved the final manuscript.

## CONFLICT OF INTEREST STATAMENT

The authors declare no conflict of interest.

## ETHICS STATEMENT

Not applicable.

## Data Availability

Not applicable.
